# Use it and/or lose it—experience effects on brain remodeling across time after stroke

**DOI:** 10.3389/fnhum.2014.00379

**Published:** 2014-06-27

**Authors:** Rachel P. Allred, Soo Young Kim, Theresa A. Jones

**Affiliations:** ^1^Department of Psychology and Institute for Neuroscience, University of Texas at AustinAustin, TX, USA; ^2^Department of Integrative Biology, University of California BerkeleyBerkeley, CA, USA

**Keywords:** upper extremity function, restorative plasticity, motor skill learning, learned non-use, motor cortex

## Abstract

The process of brain remodeling after stroke is time- and neural activity-dependent, and the latter makes it inherently sensitive to behavioral experiences. This generally supports targeting early dynamic periods of post-stroke neural remodeling with rehabilitative training (RT). However, the specific neural events that optimize RT effects are unclear and, as such, cannot be precisely targeted. Here we review evidence for, potential mechanisms of, and ongoing knowledge gaps surrounding time-sensitivities in RT efficacy, with a focus on findings from animal models of upper extremity RT. The reorganization of neural connectivity after stroke is a complex multiphasic process interacting with glial and vascular changes. Behavioral manipulations can impact numerous elements of this process to affect function. RT efficacy varies both with onset time and its timing relative to the development of compensatory strategies with the less-affected (nonparetic) hand. Earlier RT may not only capitalize on a dynamic period of brain remodeling but also counter a tendency for compensatory strategies to stamp-in suboptimal reorganization patterns. However, there is considerable variability across injuries and individuals in brain remodeling responses, and some early behavioral manipulations worsen function. The optimal timing of RT may remain unpredictable without clarification of the cellular events underlying time-sensitivities in its effects.

## Introduction

Stroke is a leading cause of chronic disability worldwide (Johnston et al., 2009). Upper extremity (hand and arm) impairments are especially prevalent lasting post-stroke disabilities (Lai et al., 2002; Kwakkel et al., 2003). Compensatory reliance on the nonparetic hand exacerbates impairments in the paretic side by encouraging its disuse (i.e., “learned nonuse,” Taub et al., 2006). Motor rehabilitative training (RT) approaches are the main tools for treating these impairments, but they are typically insufficient to normalize function. A better understanding of the mechanisms of RT efficacy could help optimize its therapeutic potential.

Ischemic injury triggers prolonged periods of neuroanatomical reorganization (Li and Carmichael, 2006; Wieloch and Nikolich, 2006; Cheatwood et al., 2008). This reorganization unfolds over months or longer, but is particularly dynamic early after stroke (Anderson et al., 1986; Carmichael, 2006; Murphy and Corbett, 2009). There are likely to be windows of opportunity for driving functionally useful brain remodeling with RT, as well as windows of vulnerability for promoting suboptimal neural changes. When is early enough? When is it safe? What should be done in these windows? The answers to these questions remain unclear. Considerable variability in neural remodeling time courses can be expected between individuals and across brain regions (e.g., Hsu and Jones, 2006; Krakauer, 2007; Riley et al., 2011). Furthermore, earlier is not better for everything. Peri-infarct tissue is vulnerable to use-dependent excitotoxicity in very early periods (Humm et al., 1998) and there is potential to ingrain maladaptive behavioral strategies (Allred and Jones, 2008a,b; Jones and Jefferson, 2011).

Motor RT relies on mechanisms of skill learning, as does compensatory learning with the nonparetic hand. In intact brains, manual skill learning depends on practice-dependent synaptic structural and functional reorganization of motor cortex (Monfils et al., 2005; Kleim et al., 2006; Xu et al., 2009; Dayan and Cohen, 2011). These learning mechanisms are likely to interact with regenerative responses to stroke, many elements of which are sensitive to behavioral manipulations, as reviewed previously (Jones and Adkins, 2010). Optimally timing and tailoring RT requires a better understanding of how it interacts with post-stroke remodeling processes as they unfold over time. Below we review a framework for understanding these interactions, progress in unraveling them and ongoing knowledge gaps surrounding time-sensitivities for experience-driven plasticity after stroke.

## A developmental framework for understanding sensitive time windows after stroke

Greenough et al. (1987) introduced the term “experience-expectant plasticity” to refer to the role of experience in brain development during early sensitive periods. The developing brain depends on external stimuli to shape neural circuitry patterns via mechanisms of synaptic competition, in which the most effectively activated neural connections are selectively maintained and matured, and those less well activated are eliminated (Black et al., 1997; Jones et al., 1998). A well-known example is the maturation of ocular dominance columns in visual cortex, which is driven by competitive activity of inputs from either eye. In the absence of visual stimulation of one eye, thalamocortical afferents of the remaining eye claim a disproportionate share of cortical territory, a pattern that is difficult to reverse (Hubel and Wiesel, 1965; Berardi et al., 2003; Wright and Bourke, 2013). This developmental process is contrasted with “experience-dependent” plasticity, i.e., the mechanism of learning. The two processes have overlapping cellular mechanisms, but vary in the magnitude and persistence of brain changes instigated by them (e.g., Zuo et al., 2005; Xu et al., 2009; Yu et al., 2013). In essence, experience-expectant plasticity establishes the major connectivity patterns of the brain and experience-dependent plasticity continuously refines this connectivity across the lifespan.

Mechanisms of experience-*dependent* plasticity clearly contribute to post-stroke brain reorganization (Williams et al., 2006; Kerr et al., 2011) and to the efficacy of RT (Nudo, 2003; Adkins et al., 2006), and they should be able to do so at any time. An unresolved question is to what extent early neural remodeling events after stroke rely on experience-*expectant* mechanisms resembling those of brain development. The regenerative responses to stroke are highly sensitive to behavioral manipulations (Jones and Adkins, 2010). The early pro-growth environment is reminiscent of development (Cramer and Chopp, 2000; Carmichael, 2006; Murphy and Corbett, 2009) and some neural restructuring events resemble those typical of brain development (Jones and Jefferson, 2011). To the degree that these responses also behave in an experience-expectant manner, one would predict early periods after stroke in which it is not only (1) relatively easy to drive neural remodeling into functionally beneficial directions using manipulations of experience and neural activity, but also (2) easy for any experiences that dominate the time window to stamp in suboptimal or maladaptive circuitry patterns that are difficult to reverse.

The first prediction above is reasonably supported, though there are still knowledge gaps that hamper its usefulness for clinical decisions, as described below. The second prediction has received less attention, but its potential implications seem equally important (Jones et al., 2013). Even with early interventions, most of the experiences of stroke survivors occur outside of the treatment context (Bernhardt et al., 2004, 2007; West and Bernhardt, 2013), creating a potential for these experiences to dominate reorganizational patterns. The existence of experience-expectant mechanisms after stroke would also raise the possibility of facilitating RT with treatments that prolong or reinstate these mechanisms (e.g., as demonstrated in visual system by Morishita and Hensch, 2008).

It is reasonable to draw upon brain development to understand brain reorganization after stroke, as cellular mechanisms for growing and re-growing neural connections overlap. However, unlike development, the adult post-stroke brain must remodel in a matrix of mature, dying, traumatized and dysfunctional structure. Stroke damages glia and vascular cells, as well as neurons, and substantially alters the intricate interactions among them. The creation of new stable patterns of neural connectivity after stroke depends on the coordinated plasticity of neurons, glia and vasculature.

## Neural, glial, and vascular remodeling: moving targets for neurorehabilitation

The loss of neurons in the core of ischemic injury leaves connected regions partially denervated and efferent neurons stripped of postsynaptic targets. The counteroffensive is the induction of a growth permissive environment that promotes axonal sprouting, synaptogenesis and dendritic remodeling (Kelley and Steward, 1997; Carmichael, 2006; Brown et al., 2010). Synapse densities around an infarct decline and then recover over time to varying degrees depending on proximity to the infarct core (Brown et al., 2008; Sigler and Murphy, 2010). Remaining projections to denervated regions sprout collateral axons and form new synapses (Cotman and Anderson, 1988; McNeill et al., 2003; Dancause et al., 2005). The axons that most prominently contribute to reinnervation tend to be the most abundant (Raisman and Field, 1990) and the most active (in firing) of the surviving projections (Carmichael and Chesselet, 2002; Carmichael, 2003; Cesa and Strata, 2005; Brus-Ramer et al., 2007). The latter property helps make the remodeling processes sensitive to manipulations of neural activity (Brus-Ramer et al., 2007; Adkins et al., 2008; Carmel et al., 2010) and behavior (Jones and Jefferson, 2011; Overman et al., 2012). There are also persistent alterations in excitatory and inhibitory activity patterns that present potential targets for combining RT with other treatments (Carmichael, 2012; Zeiler et al., 2013).

Post-ischemic reactions of neurons, astrocytes and vasculature are tightly coordinated. For example, factors expressed by glia and neurons stimulate the formation of blood vessels, and new vessels release neural growth and survival factors (Wieloch and Nikolich, 2006; Hermann and Chopp, 2012). Glia have diverse roles in mediating neuroregenerative responses (Kelley and Steward, 1997; Mack and Wolburg, 2013). Astrocytes are intricately involved in synaptic plasticity (Murai et al., 2003; Haber et al., 2006; Eroglu and Barres, 2010). Astrocytes release thrombospondins to promote synapse formation (Christopherson et al., 2005; Eroglu et al., 2009), cholesterol to promote synapse maturation (Mauch et al., 2001; Goritz et al., 2005) and D-serine to regulate synaptic potentiation and depression (Panatier et al., 2006). Astrocytic behavior is neural activity- and experience-dependent (Jones and Greenough, 2002; Theodosis et al., 2008), e.g., astrocytic reactions to denervation in motor cortex are elevated by forced forelimb use (Bury et al., 2000). After cortical infarcts, quantities of perisynaptic astrocytes and synapses vary together with injury severity (Kim and Jones, 2010), and behavioral outcome is altered by pharmacological manipulation of astrocytic glutamate transport (Kim and Jones, 2013).

There are multiphasic vascular responses after stroke. Ischemic stroke results in expanses of reduced cerebral blood flow (CBF) and capillary density (Gjedde et al., 1990; Anderson et al., 1999; el Zoppo and Mabuchi, 2003), as well as major elevations in pro-angiogenic factors (Zhang and Chopp, 2002; Hayashi et al., 2003; Carmichael, 2006; Beck and Plate, 2009), the levels of which are predictive of functional outcome in stroke patients (Slevin et al., 2000; Sobrino et al., 2007). Angiogenic microenvironments also are supportive of neurogenesis (Ohab et al., 2006). However, new vessels tend to be transient and leaky (Yu et al., 2007; Hayward et al., 2011), and there is a variable degree of recovery of CBF and vessel densities in humans (Gjedde et al., 1990; Krupinski et al., 1994; Szpak et al., 1999) and rodent models (Marti et al., 2000; Biernaskie et al., 2001; Lin et al., 2008; Mostany et al., 2011). Because sufficient blood flow is essential for activity-dependent neural remodeling, RT efficacy could depend on the success of vascular remodeling, and it might promote or accelerate it depending on its timing. For example, sensory stimulation starting 3 days after cortical ischemia promotes angiogenesis and CBF recovery (Whitaker et al., 2007).

Vascular and glial responses to injury and to behavioral experience are potentially major sources of variability in RT efficacy and its optimal timing. For example, time courses and magnitudes of astrocytic and vascular reactions to injury are altered with age (Gao et al., 2009; Brown and Thore, 2011; Popa-Wagner et al., 2011), injury severity (Kim and Jones, 2010) and diabetes (Prakash et al., 2013; Tennant and Brown, 2013). Neuroregeneration time courses and magnitudes also vary with age (Anderson et al., 1986), injury severity (Kim and Jones, 2010), injury modality (Napieralski et al., 1996; Phillips and Reeves, 2001; Voorhies and Jones, 2002; Jones et al., 2012) and other conditions (Hermann and Chopp, 2012). Thus, while there are many potential targets for treatment in stages of neurogliavascular remodeling after stroke, there is also much potential for variability in the optimal timing of these treatments.

## Earlier can be much better for rehabilitative treatments

**Figure 1:**
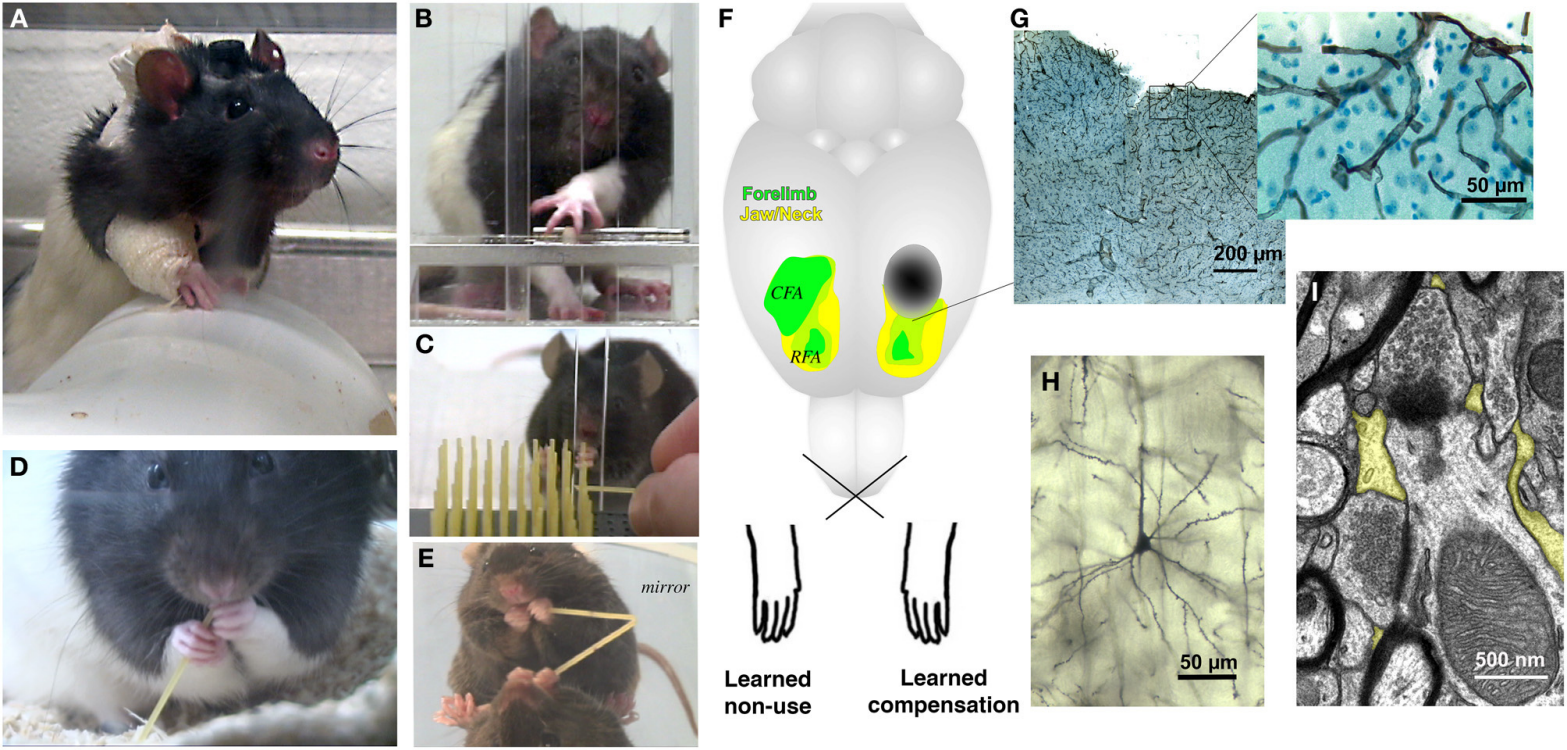
**Rodent models of upper extremity impairments after stroke used to study forelimb experience effects on brain and behavioral outcomes**. Examples of behavioral manipulations in rats and mice include **(A)** forelimb constraint, used to force greater use of the paretic limb, **(B,C)** skilled reaching tasks, used to model both rehabilitative training (RT) focused on the paretic limb and compensatory skill learning with the nonparetic limb and, **(D,E)** pasta handling tasks, used to provide coordinated bimanual experience. **(F)** Approximate motor cortical infarct location (dark oval) used in several studies, as shown relative to head (yellow) and forelimb (green) movement representation regions of motor cortex. The caudal forelimb area (CFA) is in primary motor cortex and the rostral forelimb area (RFA) is in premotor/supplementary motor cortex. Motor cortical samples showing **(G)** vasculature (collagen IV immunolabeled), **(H)** a pyramidal neuron dendritic arbor (Golgi stained) and **(I)** synapses surrounded by peri-synaptic astrocytic processes (yellow highlights). The functional efficacy of motor RT has been linked with the reorganization of movement representations in peri-infarct motor cortex, but the influence of RT over time on the remodeling of surviving neurons, glia and vasculature has not yet been well examined.

Motor RT after stroke can drive structural and functional reorganization of the injured motor cortex of humans (Taub et al., 2003; Mark et al., 2006; Dong et al., 2007; Sterling et al., 2013) and other animals (Jones et al., 1999; Biernaskie and Corbett, 2001; Frost et al., 2003; Dancause et al., 2005). In animal models, training the paretic limb in skilled reaching after cortical infarcts (Figure 1) increases its movement representation area (Castro-Alamancos and Borrel, 1995; Nudo et al., 1996) and synaptic densities (Adkins et al., 2008) in residual motor cortex of the injured hemisphere. Blocking the reorganization prevents the functional gains (Ramanathan et al., 2006). In the absence of RT, representations of the paretic limb are reduced, even well outside of infarct borders (Nudo et al., 1996).

Several studies support that RT is more effective if initiated earlier after stroke. RT beginning within 1 week of motor cortical infarcts in monkeys spares the paretic hand representation in motor cortex compared with controls (Nudo et al., 1996), but this effect is lost if training is delayed until 30 days post-infarct (Barbay et al., 2006). In rats, greater improvements in the paretic forelimb, and less compensatory reliance on the nonparetic limb, result from RT initiated within 5, vs. 14 or 30, days post-ischemia (Biernaskie et al., 2004). In humans, early (within 4 days post-stroke) interventions are associated with reduced disability at the time of hospital discharge compared with later interventions (Matsui et al., 2010). Patients receiving RT within 1 month post-stroke have greater functional improvements and require shorter RT duration to achieve them compared to those with delayed RT (Salter et al., 2006). Constraint induced movement therapy (CIMT), initiated within 3–9 months post-stroke enhances performance in several fine motor tasks compared to delayed (>9 months) CIMT (Lang et al., 2013).

In the studies above, RT timing was a categorical variable (earlier vs. later, Table 1), as is logical for determining if timing matters at all, but this does not lend precise information to the question of when, exactly, is optimal for RT onset. We also lack a precise understanding of the brain mechanisms of these time sensitivities. Our present understanding of RT mechanisms is based primarily on endpoint measures. We lack knowledge of how RT interacts with neuroremodeling responses as they unfold over time, and of the roles of vascular and glial plasticity in RT efficacy.

**Table 1:** **Time-sensitive effects of behavioral manipulations on functional outcome after brain damage in animal models**.

**Post-injury experience (onset time)**			**Functional outcome**	**References**
**Very early**	**Early**	**Delayed**		
	Motor RT (Day 5–7)		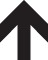	Nudo et al., 1996; Biernaskie et al., 2004
		Motor RT (Day 14)	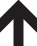	Biernaskie et al., 2004
	NPT (Day 5–7)	Motor RT (Day17–22)	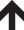	Allred et al., 2005, 2010; Allred and Jones, 2008b; Kerr et al., 2013
	NPT (Day 5–7)		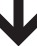	Allred et al., 2005, 2010; Allred and Jones, 2008b; Kerr et al., 2013; Maclellan et al., 2013
Exercise (Day 0)			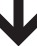	Griesbach et al., 2004
		Exercise (Day 14)	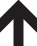	Griesbach et al., 2004
	Cognitive RT (Day 7)		**=**	Mala et al., 2012
		Cognitive RT (Day 21)	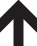	Mala et al., 2012
CNP (immediate)			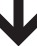	Kozlowski et al., 1996; Humm et al., 1998
	CNP (≥ Day 7)		**=**	Kozlowski et al., 1996; Humm et al., 1998
	CNP + motor RT (Day 7)		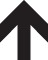	DeBow et al., 2003

*Onset time is relative to the time of injury. Behavioral manipulations continued for days to weeks after onset. Functional outcome direction is relative to no-behavioral-manipulation-controls with the same injury. RT, rehabilitative training; NPT, nonparetic limb training; CNP, constraint of the nonparetic limb*.

## Earlier is not better for everything

Schallert and colleagues were the first to discover that forced use of a paretic limb, via constraint of the nonparetic limb, can be detrimental to functional outcome if done too early (Schallert et al., 2003). In rats, forelimb impairments are worsened, and injury size increased, by constraining the nonparetic limb for 2 weeks beginning immediately after motor cortical lesions (Kozlowski et al., 1996; see also Risedal et al., 1999; Farrell et al., 2001). If constraint is delayed for 7 days, there is no detrimental effect (Humm et al., 1998). These constraint manipulations were dissimilar to the clinical application of CIMT, e.g., rats did not engage in RT and the constraint was continuous (24 h/day). In contrast, RT efficacy is improved by its combination with less intensive constraint (8 h/day) beginning 7 days after intracerbral hemorrhage in rats (DeBow et al., 2003). In humans, high intensity CIMT when initiated very early (~10 days) after stroke lessens functional improvement compared with lower intensity treatments (Dromerick et al., 2009).

Early intense exercise also can also be detrimental in rodent models of traumatic brain injury. Voluntary wheel running enhances cognitive performance if initiated *after* an acute (0–6 days) post-injury time period. However, exercise during the acute period impairs cognitive performance and prevents the normally seen up-regulation of BDNF (Griesbach et al., 2004, 2007; Griesbach, 2011).

Together, these findings support that *highly intense* physical activity *very* early after injury onset can be risky. We know of no evidence that less intense activity is detrimental. However, some types of RT might benefit from a delay, e.g., to allow resolution of metabolic dysfunction or target specific remodeling stages. Consistent with this possibility, intense cognitive training in rats is effective if it is initiated at 30 days, but not at 10 days, after hippocampal system lesions (Mala et al., 2012).

## Timing-dependencies—effects of learning to compensate with the nonparetic limb

The typical response to upper extremity impairments is to learn to rely on the better functioning limb to perform daily activities. This compensatory strategy contributes to learned nonuse of the paretic side (Taub et al., 2006) and, because it begins early after stroke, it is also likely to interact with neural remodeling events. We've studied this in rodents with motor cortical infarcts, using training on reaching tasks to examine effects of skill learning with either forelimb (Jones et al., 2013). Skill training of the nonparetic forelimb (NPT) increases dendritic growth in the contralesional cortex, but this appears not to benefit the paretic limb (Jones and Jefferson, 2011). NPT also exacerbates disuse of the paretic forelimb, impairs the efficacy of subsequent paretic limb RT (Allred et al., 2005, 2010; Kerr et al., 2013) and reduces RT-driven neuronal activation of peri-lesion cortex (Allred and Jones, 2008a,b). Thus, NPT alters a neural substrate for RT efficacy. Maclellan et al. (2013) found that paretic limb function was worsened even when tested 30 days after an earlier period of NPT. Bilateral and unskilled limb use are not detrimental to paretic limb function, but learning new unimanual skills with the nonparetic limb is detrimental, at least after motor cortical infarcts (Allred and Jones, 2008a).

The influence of the nonparetic limb could vary with infarct territories. The disruptive effects of NPT depend on contralesional motor cortex and its transcallosal projections. They are blocked by callosal transections and absent after bilateral motor cortical lesions (Allred et al., 2010). Thus, injuries that leave little remaining territory for transcallosal projections are potentially immune from maladaptive effects of compensating with the nonparetic limb.

These findings suggest that experiences of the nonparetic body side may contribute to abnormal interhemispheric interactions after stroke (Murase et al., 2004; Calautti et al., 2010). They also indicate that RT efficacy can vary, not only with its timing after stroke, but also its timing relative to the development of compensatory skills with the nonparetic body side.

## Conclusions

There are clearly early sensitive periods after stroke for the influence of RT and other behavioral experiences on functional outcome. It seems reasonable to assume that the early dynamic period of neural remodeling contribute to these time-sensitivities, but the remodeling process is complex and multiphasic, and the events or stages within it that are most important for RT efficacy have yet to be identified. For example, RT efficacy might benefit from coinciding with early stages of axonal sprouting, so that it shapes patterns of synaptic re-connectivity and effectively competes with maladaptive compensatory strategies in doing so. It could also depend on whether it is timed to coincide with stages of new vessel formation and/or stabilization, so that it can benefit from blood flow recovery or help promote it. These and other possibilities have yet to be directly tested, but it is feasible to do so in animal models of chronic stroke (Figure 1). It is also possible that, once events that contribute to heightened sensitivity to RT are identified, imaging or other assays could be used to reveal them in clinical populations. This could be essential to efforts to optimize RT, because the cellular conditions that create sensitive windows are likely to vary in time and magnitude with brain region, age, injuries and premorbid conditions. An “early” that is reliably *best* for RT in a reasonable portion of the clinical stroke population could be elusive in the realm of time, as measured by hours and days, but there is potential for it to be found within stages of sequential brain events. Knowledge of the events that create sensitive windows for experience-driven plasticity after stroke also could lead to treatments that promote these windows when they are deficient or reopen them after they have passed.

### Conflict of interest statement

The authors declare that the research was conducted in the absence of any commercial or financial relationships that could be construed as a potential conflict of interest.
